# *N*-Heterocyclic Carbenes and Their Metal Complexes Based on Histidine and Histamine Derivatives of Bacteriopurpurinimide for the Combined Chemo- and Photodynamic Therapy of Cancer

**DOI:** 10.3390/ijms232415776

**Published:** 2022-12-12

**Authors:** Sergey Tikhonov, Natalia Morozova, Anna Plutinskaya, Ekaterina Plotnikova, Andrey Pankratov, Olga Abramova, Ekaterina Diachkova, Yuriy Vasil’ev, Mikhail Grin

**Affiliations:** 1 Department of Chemistry and Technology of Biologically Active Compounds, Medicinal and Organic Chemistry, Institute of Fine Chemical Technologies, MIREA-Russian Technological University, 86 Vernadsky Avenue, 119571 Moscow, Russia; 2 P. Hertsen Moscow Oncology Research Institute—Branch of the National Medical Research Radiological Centre of the Ministry of Health of the Russian Federation, 2nd Botkinsky pr., 3, 125284 Moscow, Russia; 3 A. Tsyb Medical Radiological Research Center—Branch of the National Medical Research Radiological Center of the Ministry of Health of the Russian Federation (A. Tsyb MRRC), 249031 Obninsk, Russia; 4 Department of Oral Surgery of Borovsky Institute of Dentistry, I.M. Sechenov First Moscow State Medical University (Sechenov University), Trubetskaya St. Bldg. 8\2, 119435 Moscow, Russia; 5 Department of Operative Surgery and Topographic Anatomy, I.M. Sechenov First Moscow State Medical University (Sechenov University), Trubetskaya St. Bldg. 8\2, 119435 Moscow, Russia

**Keywords:** PDT, bacteriopurpurinimide, chemotherapy, cancer, gold complexes

## Abstract

Photodynamic therapy (PDT) is currently regarded as a promising method for the treatment of oncological diseases. However, it involves a number of limitations related to the specific features of the method and the specific characteristics of photosensitizer molecules, including tumor hypoxia, small depth of light penetration into the tumor tissue, and low accumulation sensitivity. These drawbacks can be overcome by combining PDT with other treatment methods, for example, chemotherapy. In this work, we were the first to obtain agents that contain bacteriopurpurinimide as a photodynamic subunit and complexes of gold(I) that implement the chemotherapy effect. To bind the latter agents, *N*-heterocyclic carbenes (NHC) based on histidine and histamine were obtained. We considered alternative techniques for synthesizing the target conjugates and selected an optimal one that enabled the production of preparative amounts for biological assays. In vitro studies showed that all the compounds obtained exhibited high photoinduced activity. The C-donor Au(I) complexes exhibited the maximum specific activity at longer incubation times compared to the other derivatives, both under exposure to light and without irradiation. In in vivo studies, the presence of histamine in the NHC-derivative of dipropoxy-BPI (**7b**) had no significant effect on its antitumor action, whereas the Au(I) metal complex of histamine NHC-derivative with BPI (**8b**) resulted in enhanced antitumor activity and in an increased number of remissions after photodynamic treatment.

## 1. Introduction

Various protocols are currently available for the treatment of oncological diseases, including those that combine photodynamic and chemotherapy [1]. This approach makes it possible to enhance the treatment efficiency and reduce the dose of chemotherapeutic agents and hence, mitigate the side effects of chemotherapy. Photolon was used as a photosensitizer for photodynamic therapy, and cisplatin was employed for chemotherapy. The most efficient treatment procedure was found to involve photodynamic therapy with irradiation in 2 h after administering Photolon and administering cisplatin in one and four days after photodynamic therapy with a total chemotherapeutic agent dose of 2.5 mg/kg. By the end of the study, complete regression was observed in 88.9% of rats, and inhibition of tumor growth was found in 99.9% of the animals. Based on the results of that study, the authors concluded that the combination of photodynamic therapy with cisplatin chemotherapy was a more efficient treatment method compared with each therapy alone [2].

The combined chemo- and photodynamic therapy based on a drug molecule containing both a photodynamic and a chemotherapeutic agent is advantageous in comparison with a single-agent therapy since it provides the targeted delivery of chemotherapeutic agents into the tumor due to the tumor affinity of photosensitizers of porphyrin type and, as a consequence, a reduction in the therapeutic dose of the chemotherapeutic agent, which, in turn, diminishes the system toxicity for the organism [3]. Combining various therapeutic effects in a single drug may provide a path to the solution of the multiple drug resistance problem, where the PDT destroys the resistant cancer cells that survived the chemotherapy [4].

Another approach to the creation of combined drugs is the preparation of self-assembling systems based on liposomes, nanoparticles, and other nanocarriers of various natures. Such systems can solve problems with the medicinal agents’ delivery to the area of interest and carry multi actions agents. Recently, carriers based on liposomes and peptide nanoparticles have become widely used for the implementation of combined treatment methods along with metal nanoparticles. These structures showed better results in vivo compared to non-nanostructured analogs. A huge amount of different combinations of nanocarriers, photosensitizers, and chemotherapeutic agents can be found in the literature over the world [5,6,7].

Bi- and multifunctional agents based on *N*-heterocyclic carbenes (NHC) may be employed for this goal. NHC-based metal complexes intended for such cell-based targets as mitochondria, DNA, and proteins of the antioxidant cell protection may be promising combined-action agents [8]. Numerous academic studies are now focused on the use of *N*-heterocyclic carbenes as an alternative to thiols, amines, and pyridines for binding with complexes of gold, platinum, and other metals. The main feature of *N*-heterocyclic carbenes is that their structure comprises a C-donor moiety. The latter can be represented by the imidazole ring of histidine and histamine that is involved in the formation of complexes with metals and is used to functionalize nanoparticles [9]. Many NHC-metal complexes have already demonstrated their biological activity [10,11], including antimicrobial [12] and antitumor effects [13,14].

Histidine, an amino acid, and histamine, a biogenic amine obtained on its basis, are part of various drugs and are used as vectors for the delivery of chemotherapeutic agents and as precursors for synthesizing histamine receptor inhibitors [14]. Histamine is biosynthesized in the organism by L-histidine decarboxylase. The discovery of histamine receptors in various tissues gave an understanding of numerous physiological and pathophysiological processes. A special role in carcinogenesis is attributed to H3 and H4 histamine receptors [15]. The use of agonists and antagonists of these receptors designed based on histamine in antitumor therapy may result in a significant enhancement in the selectivity of the delivery of chemotherapeutic agents to a zone of interest. Studies on antitumor immunity and the development of the immunotherapy of tumors provided an understanding of the special role of the H3 and H4 receptors in the development of cancer. Brandes has shown that intracellular histamine performs as a secondary messenger binding with human P450 cytochrome (CYP450) and thus suppressing the proliferation of lymphocytes and hemopoietic precursors, which results in a reduction in the efficiency of immunity toward cancer cells [16].

The platinum-group metals, gold, tin, gadolinium, technetium, and copper, are the most promising metals for biomedical applications, including oncology [17]. Gold(I) complexes have some advantages compared with the other metals listed above, as confirmed by extensive studies on the therapeutic use of gold complexes as antitumor agents [18]. For example, a complex of thioglucopyranose and gold(I) triethyl phosphine, better known as auranofin, was very efficient in clinical practice for treating arthritis [19]. One of the targets of gold(I) complexes is thioredoxin reductase (TrxR), an enzyme of the cellular antioxidant system, which protects the cell against the excessive formation of reactive oxygen species (RAS). Tumor cells contain a high level of TrxR, so inhibitors of this enzyme can be potential candidates for the treatment of oncological diseases [20,21].

It has been shown that complexes of gold(I) with *N*-heterocyclic carbenes based on histidine and imidazole exhibit high cytotoxicity toward the cisplatin-resistant lineage of A2780cis cells. In addition, in vivo studies have shown that NHC complexes of gold(I) are more efficient in the case of one-off intake compared with administering cisplatin as a course [22].

From the chemical perspective, an advantage of gold(I) complexes is that it is possible to incorporate two different ligands that can be used to control the biological effect in the desired way. For example, if sugars are used as one of the ligands, a drug with enhanced selectivity of accumulation in a tumor can be obtained [23]. Variations of substituents may cause an increase in the stability of complexes in biological environments and change the intracellular targets while making the synthesis more complicated and reducing biological activity [24].

Photosensitizers (PS) based on bacteriochlorophyll *a* derivatives, in particular, bacteriopurpurinimides (BPI) that absorb radiation in the 800 nm range, are promising agents for photodynamic therapy (PDT), which we are actively studying, including the synthesis of amino acid derivatives of BPI and their metal complexes as agents with combined antitumor action [25,26].

Several techniques for the synthesis of metal complexes of natural chlorins by means of chelation on the macrocycle periphery have been developed. Our research team has previously implemented two approaches for assembling the target molecules. The first option includes the modification of functional groups or residues of bioactive molecules on the macrocycle periphery. The other approach aims at creating a metal complex of an amino acid followed by binding it to bacteriochlorin [26,27,28,29].

We were the first to synthesize bacteriochlorin-series photosensitizers that contain histidine and histamine molecules, *N*-heterocyclic carbenes of histidine and histamine, and carbon-donor metal complexes of Au(I) on their basis.

## 2. Results and Discussion

### 2.1. Chemistry

**Figure 1:**
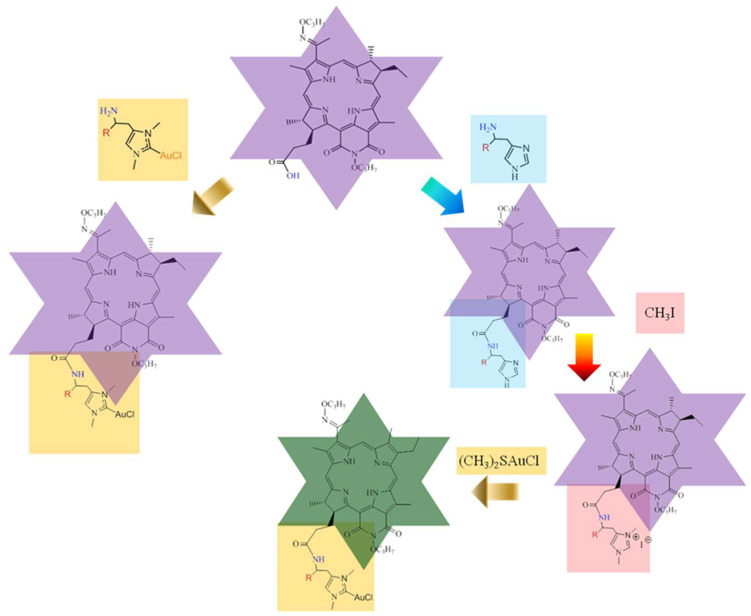
Strategy for the synthesis of conjugates.

The target conjugates can be synthesized in two ways. The first approach is based on the synthesis of gold complexes with *N*-heterocyclic carbenes pre-synthesized from histidine or histamine methyl ester, followed by binding to a PS molecule. The second approach involves a stage-by-stage assembly of the complex based on a PS molecule, which includes binding of histidine or histamine methyl ester to a PS molecule, methylation of the imidazole moiety, and finally, the addition of gold to the carbene carbon atom (Figure 1).

Within the first approach, mono-Boc protected histidine **1a** and histamine **1b** were synthesized for subsequent synthesis of Au(I) metal complexes with an *N*-heterocyclic carbene based on *N*-methylated imidazole [30].

**Scheme 1:**
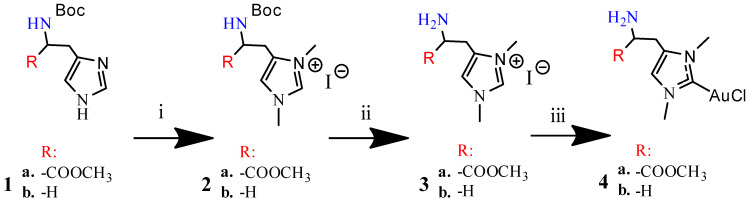
Reagents and conditions: (i): CH_3_I 8% in CH_3_CN, K_2_CO_3_, 70 °C, 16 h, (ii): HCl in dioxane, 24 h (iii): 1. CH_2_Cl_2_, Ag_2_O, dark, 1 h; 2. (CH_3_)_2_SAuCl, dark, 1 h.

It was found, however, that deblocking the Boc-protected amino group after binding the gold complex was accompanied by the elimination of the latter from the carbene carbon atom. Therefore, the sequence of reactions was changed, and deblocking was performed before binding the gold complex, as shown in Scheme 1. A solution of HCl in dioxane was found to be the optimal agent for the removal of Boc-protection from NHC derivatives of histidine and histamine (Scheme 1). The gold complexes **4a** and **4b** thus obtained were characterized using ^1^H NMR spectra, which demonstrated the absence of the carbene proton. The metal complexes had much better solubility in aqueous solutions than metal-free NHC derivatives of histidine **3a** and histamine **3b** and remained stable for a long time.

**Scheme 2:**
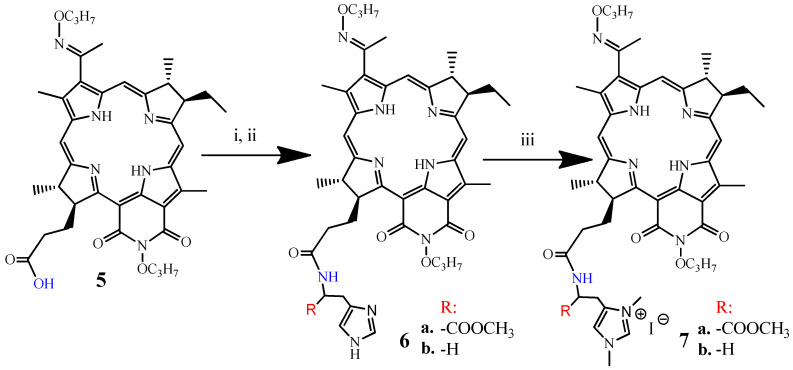
Reagents and conditions: (i): 1. CH_2_Cl_2_, EDC, NHS, 0 °C, 1 h, 2. histamine hydrochloride, Et_3_N, 36 h, (ii): 1. CH_2_Cl_2_, EDC, NHS, 0 °C, 1 h, 2. histidine methyl ester dihydrochloride, Et_3_N, 36 h, Ar, (iii): CH_3_I 8% in CH_3_CN, K_2_CO_3_, 70 °C, Ar, 16 h.

A scheme for the synthesis of NHC derivatives of histidine and histamine by assembling on a macrocycle is shown below (Scheme 2).

The carboxyl group of the propionic residue in position 17 of the dipropoxybacteriopurpurinimide **5** macrocycle was amidated using the carbodiimide method by the reaction of the latter with histidine or histamine methyl ester to give amides **6a** and **6b**, respectively. The synthesis of the conjugate with histidine was accompanied by the formation of two products, namely, the target compound **6a** and its protonated form. The latter was formed during the storage of **6a**, apparently due to the reaction of this compound with water vapor and carbon dioxide from the air. The absorption and electronic fluorescence spectra were like those of the original PS. To obtain NHC derivatives, bacteriopurpurinimides containing histidine **6a** and histamine **6b** were refluxed in an 8 vol.% solution of CH_3_I in acetonitrile in a continuous stream of argon to prevent the oxidation of the bacteriochlorin macrocycle. The polarity of the resulting compounds strongly increased due to the presence of a cationic center, which complicated their isolation and purification. Chromatographic purification of these compounds was performed using dichloromethane: methanol system (10:1 *v*/*v*).

**Scheme 3:**
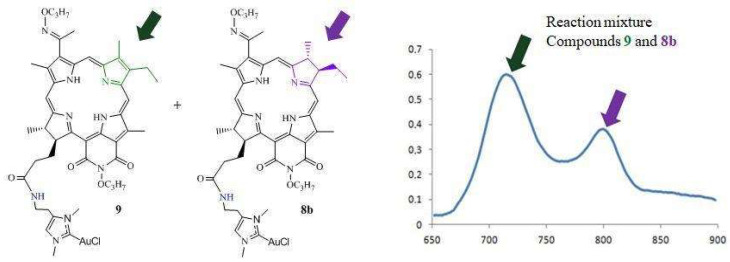
Products of the reaction of **7b** with gold(I) dimethylsulfide chloride. Green arrow—chlorine (λ = 720 nm), Purple arrow—bacteriochlorin (λ = 800 nm).

The reaction of NHC derivative **7b** with gold(I) dimethylsulfide chloride ((CH_3_)_2_SAuCl) yielded a mixture of the target metal complex **8b** with chlorin **9**, where the latter predominated. Variation of the reaction conditions and the use of Ag_2_O as the catalyst did not alter the composition of the product mixtures obtained (Scheme 3). Presumably, oxidation of the macrocycle with gold(I) occurred. This is confirmed by a gold deposit on the reaction flask, which is indicative of Au^0^ formation.

**Scheme 4:**
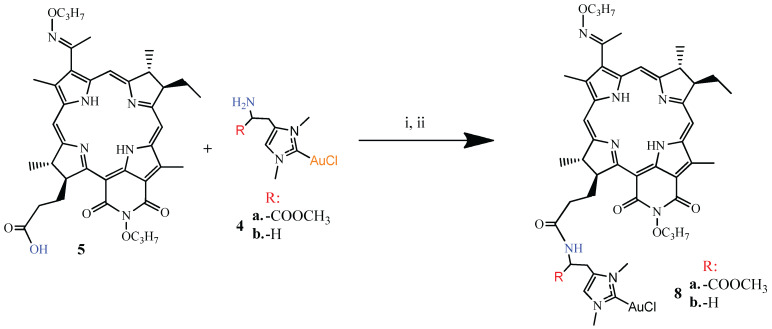
Reagents and conditions: (i): 1. CH_2_Cl_2_, EDC, NHS, 0 °C, 1 h. 2. compound **4a**, Et3N, 36 h; (ii): 1. CH_2_Cl_2_, EDC, NHS, 0 °C, 1 h, 2. compound **4b**, Et3N, 36 h, Ar.

The ambiguous pathway of the reactions while assembling the target metal complexes prompted us to use the gold(I) complexes based on NHC histidine and histamine **4a** and **4b** that we obtained previously to bind them to bacteriopurpurinimide **5**, which exhibited a high photodynamic efficiency, also obtained by our team earlier [31]. The binding was carried out by the activated ester method using 1-ethyl-3-(3-dimethylaminopropyl)carbodiimide (EDC) and N-hydroxysuccinimide (NHS). The synthesis of the target metal complexes **8a** and **8b** was monitored using thin-layer chromatography, which showed a reduction in the mobility of the reaction products compared to the dipropoxyBPI NHS ester (Scheme 4).

**Figure 2:**
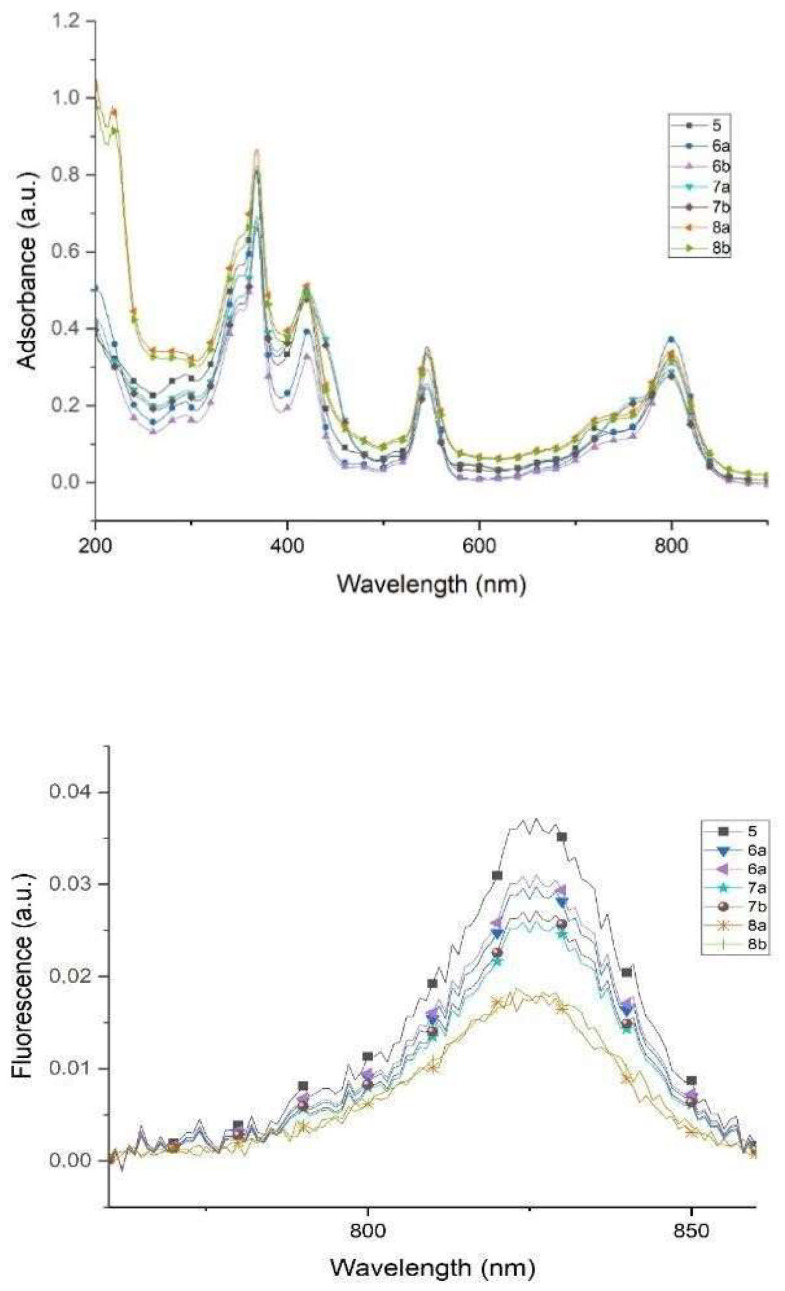
Absorption and electronic fluorescence spectra of dipropoxy-BPI 5; amino acid modifications **6a**,**b**; NHC-compounds **7a**,**b** and metal complexes **8a**,**b**.

The compounds obtained exhibited some changes in the absorption spectra and a reduction in the fluorescence intensity by 30–40% (Figure 2).

The electronic spectra of conjugates **8a** and **8b** exhibited a strong absorption line in the UV range at 220 nm, characteristic of NHC complexes of gold(I).

### 2.2. Biology

#### 2.2.1. In Vitro

The specific activity of the compounds obtained was studied on two cell lines: prostate adenocarcinoma (PC-3) with hyperexpression of histamine receptors and colon carcinoma (HCt116) with weak expression of histamine receptors. [32,33]

The activity of compounds was estimated both under light exposure (photoinduced activity) and without exposure (cytotoxic activity). Moreover, the combined effect of the compounds was studied in a two-step experiment: incubation of substances with cells for 4 h followed by exposure to light at a wavelength corresponding to the PS absorption maximum. After completion of light exposure, the plates with cells were placed in a CO_2_ incubator for 48 or 72 h to assess the cytotoxic effect realized due to the Au(I) chemotherapeutic agent.

**Figure 3:**
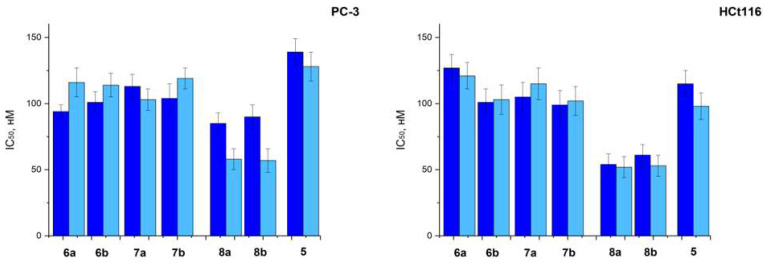
The ccombined activity of compounds in vitro as a function of incubation time after administering (dark blue columns—48 h, light blue columns—72 h).

The results are presented in Table 1 and Table 2 and in Figure 3. All the compounds exhibited highly comparable photoinduced activity against PC-3 and NST116 tumor cells. The pronounced additive effect of the PS and the gold complex was obtained for compounds **8a** and **8b**. An increase in specific activity with an increase in the incubation time to 72 h by a factor of 1.7–2.4 was revealed, which may indicate that Au(I) contributes as a chemotherapeutic agent to the efficiency of the compounds (Figure 1). The cytotoxic activity of derivatives **8a** and **8b** also increased 1.9-fold and 2.5-fold, respectively, with an increase in incubation time to 72 h (Table 2).

#### 2.2.2. In Vivo

Normalized fluorescence and fluorescence contrast.

**Figure 4:**
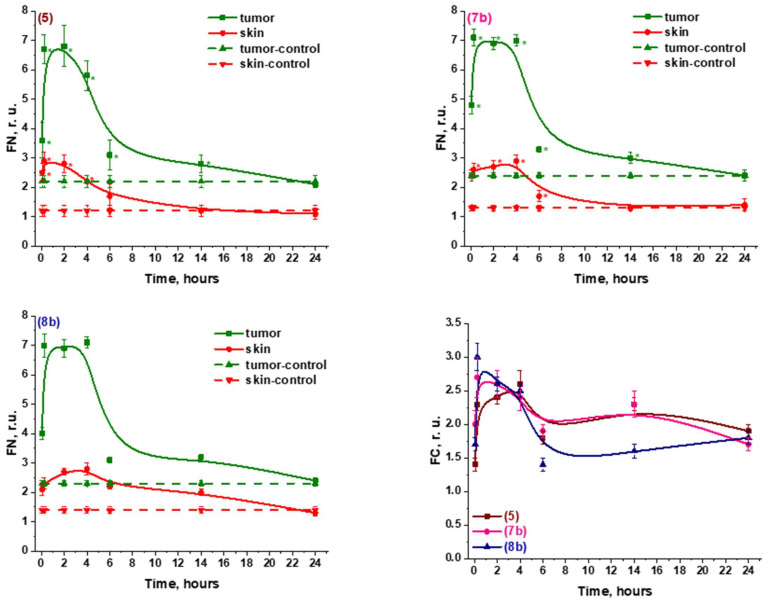
Normalized fluorescence and fluorescence contrast of the compounds in PC-3 tumor tissue and in the surrounding skin in mice, in the time range from 5 min to 24 h. Data are presented with the background values taken into account. Fluorescence was excited by He-Ne laser radiation (λmax = 633 nm). The measurements were performed in the range of 640–900 nm. *—*p* ≤ 0.05.

Based on the results obtained in in vitro tests, the NHC-derivative of dipropoxy-BPI with histamine (**7b**) and its metal complex with Au(I) (**8b**) were chosen for studying the in vivo anti-tumor efficiency in comparison with dipropoxy-BPI (**5**). The studies were performed on immunodeficient nu/nu mice with human prostate adenocarcinoma xenografts (PC-3). The results of normalized fluorescence and fluorescence contrast are shown in Figure 4. 

All the compounds were accumulated in PC-3 adenocarcinoma tissue. Normalized fluorescence of the photosensitizers was instantly detected in tumor tissue and reached the maximum values 15 min after injection. It was maintained at a high level for 2 h after injection of compound (**5**) and for 4 h in the case of compounds (**7b**) and (**8b**). The longer retention of compounds (**7b**) and (**8b**) is apparently caused by the presence of histamine in the photosensitizer (PS) molecule, which promotes binding to receptors on the surface of tumor cells (Figure 4). The elimination of the PS from the tumor and the surrounding tissues occurred 24 h after the intravenous injection.

In the surrounding skin, the PS was detected at a level slightly higher than the background values for 14 h after injection. The maximum level of fluorescence contrast (FC) was observed in 15 min to 4 h after injection and ranged from 2.3 ± 0.1 to 3.0 ± 0.2 rel. units.

#### 2.2.3. Specific Activity

**Figure 5:**
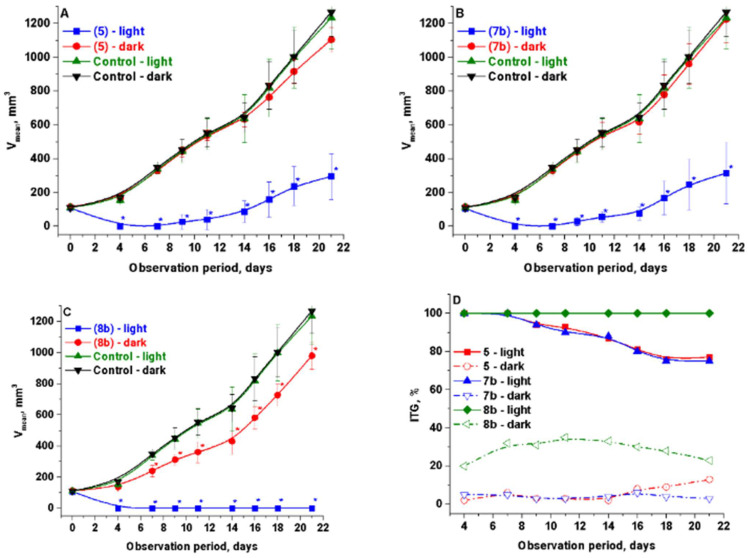

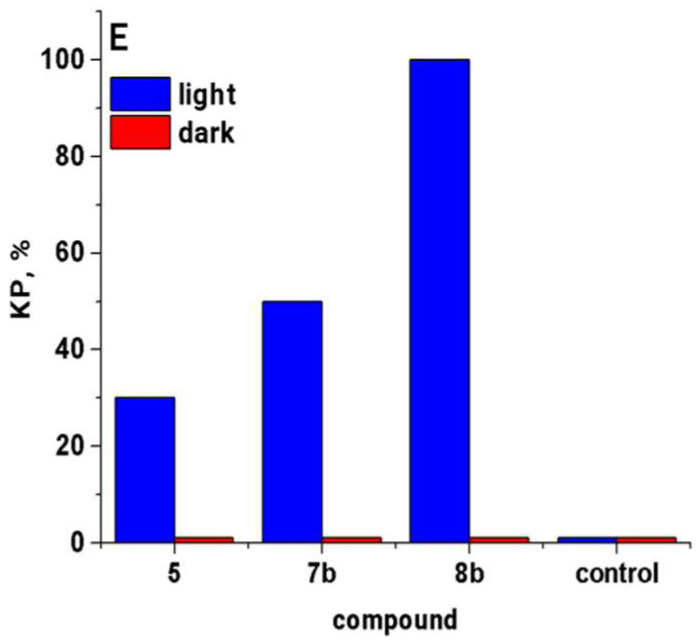
Photoinduced antitumor activity of NHC-derived dipropoxy-BPI with histamine (**7b**)—(**B**) and its metal complex with Au (I) (**8b**)—(**C**) versus dipropoxy-BPI (5)—(**A**) in mice with PC-3 adenocarcinoma xenografts. ITG was calculated in comparison to the “Control—dark” group—(**D**); KP—(**E**). The compound doses were counted as 1.0 mg/kg. The time interval between injection and irradiation lasted 15 min. Radiation source wasALHT-ELOMED laser device (Elomed, Dubna, Russia) for PDT, 810 nm, 100 mW/cm^2^ power density, 90 J/cm^2^ energy density. The animals were observed for 30 days. Animals of the control group were injected with 0.9% NaCl. *—*p* ≤ 0.05.

In the studies on immunodeficient nu/nu mice with PC-3 human prostate adenocarcinoma xenografts, the photoinduced antitumor activity of NHC-derived dipropoxy-BPI with histamine (**7b**) and its metal complex with Au(I) (**8b**) was estimated in comparison with that of dipropoxy-BPI (**5**). At the beginning of treatment, the tumor size was 110 ± 10 mm^3^. A PS was administered once, intravenously at a dose of 1.0 mg/kg, based on the active ingredient (dipropoxy-BPI). The results of these studies are presented in Figure 5. All the compounds were shown to have high antitumor activity. Compounds **7b** and **5** were shown to have similar antitumor effects: the inhibition of tumor growth (ITG) was 76–100% and 75–100%, respectively; the number of remissions (NR) was 30% and 50%, respectively. A higher antitumor effect was observed with the Au(I) metal complex **8b** as the PS: ITG = 100%, NR = 100%. On the one hand, it is due to the presence in the drug molecule of a long-wave PS (dipropoxy-BPI) modified by histamine that is capable of binding to the corresponding receptors of PC-3 adenocarcinoma, and on the other hand, to the presence of Au(I) as a chemotherapeutic agent. The use of compound **8b** in mice with PC-3 xenografts without light exposure resulted in an insignificant inhibition of tumor growth: ITG varied from 20 to 35% (the differences from the control group were biologically significant between days 7 and 21).

The compounds of bacteriochlorins with metals described earlier contain metal inside a macrocycle, or the pigment is immobilized on the surface of metal nanoparticles [34]. Opposed to the described compounds, in the conjugates obtained by us, the metal is included in an external chelating fragment (NHC-histidine and histamine). One of the advantages of such structures is the preservation of the spectral properties of the PS molecule; for example, the key PS used in the work, dipropoxy-BPI, retains the absorption λ = 800 nm when attached to the periphery of the macrocycle of the metal complex. The disadvantages of the obtained conjugates include the low solubility of the latter in polar solvents and water, which requires the use of solubilizers, including Kolliphore, Pluronic, et al.

## 3. Materials and Methods

### 3.1. Chemistry

The solvents were purified and prepared using standard procedures. Dimethylsulfidegold(I) was purchased from AcrosOrganic (Beijing, China). *O-*Propyloxime-*N-*propoxybacteriopurpurinimide (**6**) was obtained by the standard method developed in our laboratory [31]. TLC was performed using 60 Merck silica gel (Darmstadt, Germany) on 20 × 20 cm plates with a thickness of 1 mm. Mass spectra were recorded on a Bruker Ultraflex TOF/TOF mass spectrometer (Bremen, Germany) by the MALDI method using 2,5-dihydroxybenzoic acid (DHB) as a matrix, as well as on a TSQ QuantumAccess MAX triple quadrupole mass spectrometer (Waltham, MA, USA) by the ESI method. NMR spectra were recorded on a Bruker DPX-300 spectrometer (Bremen, Germany) at 25 °C in DMSO-d6 at a working frequency of 300 MHz. Residual signals of ^1^H nuclei were used to calibrate the scale. Experiments were performed according to standard Bruker methods. IR spectra were obtained on an Infralum FT-08 FT-IR spectrometer (Moscow, Russia) in KBr pellets. Fluorescence spectra were obtained on a Lumex Fluorat-02 PANORAMA spectrofluorimeter (Russia, Moscow) in ethanol at an excitation wavelength of 800 nm. Electronic absorption spectra were obtained in ethanol on an SF-2000 spectrophotometer manufactured by OKB SPEKTR (Saint Petersburg, Russia).

#### 3.1.1. Synthesis of N-boc-Histidine-NHC and N-boc-Histamine-NHC (**2a** and **2b**)

N-boc-Imidazole (2 mmol) was dissolved in acetonitrile, then CH_3_I (40 mmol, 8% in acetonitrile) and K_2_CO_3_ (18 mmol) were added to the solution. The reactions were carried out under reflux for 16 h. Then, the reaction mixture was cooled to room temperature. The solvent was removed in vacuo. The white-yellow precipitate was redissolved in CH_2_Cl_2._ The resulting suspension was filtered off. The filtrate was concentrated in vacuo. The product was recrystallized from petroleum ether.

Compound **2a**: Yield 70%, ^1^H NMR (300 MHz, CDCl_3_) δ: 1.37 (s, 9H, 3^×^CH_3_), 3.24 (t, 2H, CH_2_), 3.77 (t, 3H, -OCH_3_), 3.97 (d, 3H, 2xN-CH_3_), 5.70 (s, 1H, 5-Ar-H), 7.30 (s, 1H, 3-Ar-H), 9.79 (s, 1H, NH).

^13^C NMR (300 MHz, CDCl_3_) δ: 26.43-CH_2_; 28.18-3^×^CH_3_; 34.34-N-CH_3_; 36.73-N-CH_3_; 52.01-OCH_3_; 53.21-CH; 82.34-(CH_3_)_3_-C-; 121.68-5- Ar-C; 131.71-1-Ar-C; 137.14-3-Ar-C; 155.46-C=O; 170.64-C=O.

Compound **2b**: Yield 83%, ^1^H NMR (300 MHz, CDCl_3_) δ: 1.35 (s, 9H, 3^×^CH_3_), 2.90 (t, 2H, CH_2_), 3.39 (t, 2H, CH_2_), 3.91 (s, 3H, N-CH_3_), 3.95 (s, 3H, N-CH_3_), 5.51 (s, 1H, N-H), 7.37 (s, 1H, 5-Ar-H), 9.60 (s, 1H, 3-Ar-H).

^13^C NMR (300 MHz, CDCl_3_) δ: 24.53-CH_2_; 28.33-3^×^CH_3_; 34.39-N-CH_3_; 36.50-N-CH_3_; 38.18-CH_2_; 79.49-(CH_3_)_3_-C-; 121.11-5-Ar-C; 133.57-1-Ar-C; 136.59-3-Ar-C; 156.18-C=O.

#### 3.1.2. Synthesis of Histidine-NHC-C-AuCl complex and Histamine-NHC-C-AuCl Complex (**4a** and **4b**)

A Boc-protected NHC compound (**2a** or **2b**) (0.5 mmol) was dissolved in 5 mL HCl (2M) in dioxane, and the process was carried out for 24 h at room temperature. Deprotection was monitored by TLC. The product was isolated by removing the solvent in vacuo.

An NHC compound (**3a** or **3b**) (0.5 mmol) was dissolved in CH_2_Cl_2_, the flask was covered with a black film to protect it from light, and Ag_2_O (0.5 mmol) was then added. The reaction was carried out by stirring under an argon atmosphere at room temperature for 1 h. After that, (CH_3_)_2_SAuCl (0.5 mmol) was added to the reaction mixture. The reaction was then carried out for 1 h under the same conditions. The solvent was removed in vacuo to give pale yellow crystals. The products were recrystallized from a CH_2_Cl_2_: hexane solution.

Compound **3a**: Yield 78%, ^1^H NMR (300 MHz, CDCl_3_) δ: 1.37 (s, 9H, 3_^×^_CH_3_), 3.24 (t, 2H, CH_2_), 3.77 (t, 3H, -OCH_3_), 3.97 (d, 3H, 2xN-CH_3_), 5.70 (s, 1H, 5-Ar-H), 7.30 (s, 1H, 3-Ar-H), 9.79 (s, 1H, NH).

^13^C NMR (300 MHz, CDCl_3_) δ: 26.43-CH_2_; 28.18-3_^×^_CH_3_; 34.34-N-CH_3_; 36.73-N-CH_3_; 52.01-OCH_3_; 53.21-CH; 82.34-(CH_3_)_3_-C-; 121.68-5-Ar-C; 131.71-1-Ar-C; 137.14-3-Ar-C; 155.46-C=O; 170.64-C=O.

Compound **3b**: Yield 89%, ^1^H NMR (300 MHz, CDCl_3_) δ: 1.35 (s, 9H, 3_^×^_CH_3_), 2.90 (t, 2H, CH_2_), 3.39 (t, 2H, CH_2_), 3.91 (s, 3H, N-CH_3_), 3.95 (s, 3H, N-CH_3_), 5.51 (s, 1H, N-H), 7.37 (s, 1H, 5-Ar-H), 9.60 (s, 1H, 3-Ar-H).

^13^C NMR (300 MHz, CDCl_3_) δ: 24.53-CH_2_; 28.33-3_^×^_CH_3_; 34.39-N-CH_3_; 36.50-N-CH_3_; 38.18-CH_2_; 79.49-(CH_3_)_3_-C-; 121.11-5-Ar-C; 133.57-1-Ar-C; 136.59-3-Ar-C; 156.18-C=O.

Compound **4a**: Yield 72%, ^1^H NMR (300 MHz, CDCl_3_) δ: 1.37 (s, 9H, 3^×^CH_3_), 3.24 (t, 2H, CH_2_), 3.77 (t, 3H, -OCH_3_), 3.97 (d, 3H, 2xN-CH_3_), 5.70 (m, 1H, N-H), 7.30 (s, 1H, 3-Ar-H).

Calc. for C_9_H_15_AuN_3_ClO_2_ (%): C 25.16; H 3.52; N 9.78; Au 45.84; O 7.45; Cl 8.25. Found (%):C 25.13; H 3.55; N 9.76; Au 45.86; O 7.41; Cl 8.29.

Compound **4b**: Yield 89%, ^1^H NMR (300 MHz, CDCl_3_) δ: 1.35 (s, 9H, 3^×^CH_3_), 2.90 (t, 2H, CH_2_), 3.39 (t, 2H, CH_2_), 3.91 (s, 3H, N-CH_3_), 3.95 (s, 3H, N-CH_3_), 5.51 (s, 1H, N-H), 7.37 (s, 1H, 5-Ar-H).

Calc. for C_7_H_13_AuN_3_Cl (%): C 22.62; H 3.53; N 11.31; Au 53.00; O 0.00; Cl 9.54. Found (%): C 22.67; H 3.55; N 11.31; Au 52.95; O 0.00; Cl 9.52.

#### 3.1.3. Synthesis of DPI-Histidine and DPI-Histamine (**6a** and **6b**)

DPI (0.09 mmol) was dissolved in CH_2_Cl_2_, then EDC (0.135 mmol) and NHS (0.234 mmol) were added to this solution with stirring at 0 °C. After activation for 1 h, histidine methyl ester dihydrochloride (0.45 mmol) was added to the mixture with stirring, then Et_3_N (200 μL) was added dropwise, and the reaction was carried out under an argon atmosphere for 36 h. The solvent was removed in vacuo. The product was obtained by TLC with CH_2_Cl_2_: MeOH 20:1.

Compound **6a**: Yield 60%, ^1^H NMR (300 MHz; CDCl_3_ ppm): 8.53 (1H, s, 5-H); 8.48 (1H, s, 10-H); 8.43 (1H, bs, 7′-NH); 8.36 (1H, s, 20-H); 7.49 (1H, s, 8′-H); 7.15 (1H, s, 6′-H); 5.11 (1H, d, 1′-NH); 4.50-4.36 (4H, m, 2x-OCH_2_CH_2_CH_3_); 4.33 (1H, t, 2′-CH); 4.20-3.91 (3H, m, 7-H, 18-H, 8-H); 3.75 (3H, s, 2′-COOCH_3_); 3.55 (3H, s, 21-CH_3_); 3.41 (2H, m, 3′-CH_2_); 3.24 (3H, s, 121-CH_3_); 2.7 (3H, s, 32-CH_3_); 2.48 (6H, m, 171 CH_2_; 2x-OCH_2_CH_2_CH_3_); 2.31 (2H, m, 81-CH_2_); 1.99 (2H, m, 172-CH_2_); 1.78 (3H, d, OCH_2_CH_2_CH_3_); 1.68 (3H, d, OCH_2_CH_2_CH_3_); 1.26 (3H, s, 181-CH_3_); 1.17 (3H, t, 71-CH_3_); 1.07 (3H, t, 82-CH_3_); 0.42 (1H, bs, NH); 0.12 (1H, bs, NH).

Compound **6b**: Yield 71%, ^1^H NMR (300 MHz; CDCl_3_ ppm): 8.498 (1H, s, 5-H); 8.478 (1H, s, 10-H); 8.361 (1H, s, 20-H); 7.804 (1H, bs, 5′-CH); 7.160 (1H, s, 7′-CH); 5.040 (1H, s, 1′-NH); 4.438 (2H, t, -OCH_2_CH_2_CH_3_); 4.37 (2H, t, -OCH_2_CH_2_CH_3_); 4.20-4.00 (3H, m, 7-H, 18-H, 8-H); 3.96 (2H, m, 1′-CH_2_); 3.63 (3H, m, 21-CH_3_); 3.51 (3H, s, 121-CH_3_); 3.23 (2H, m, 3′-CH_2_); 2.70 (3H, s, 32-CH_3_); 2.32 (2H, m, 81-CH_2_); 2.08-1.92 (8H, m, 172-CH_2_; 2x-OCH_2_CH_2_CH_3_); 1.78 (3H, d, -OCH_2_CH_2_CH_3_); 1.65 (3H, d, -OCH_2_CH_2_CH_3_); 1.62 (2H, s, 171-CH_2_); 1.10 (6H, d, 181-CH_3_; 71-CH_3_); 0.86 (3H, d, 82-CH_3_); 0.38 (1H, bs, NH); 0.08 (1H, bs, NH).

#### 3.1.4. Synthesis of DPI-Histidine-NHC and DPI-Histamine-NHC (**7a** and **7b**)

DPI (0.03 mmol) was dissolved in acetonitrile, then CH_3_I (0.60 mmol, 8% in acetonitrile) and K_2_CO_3_ (0.30 mmol) were added to the solution. The reactions were carried out under reflux for 16 h under an argon atmosphere. The reaction mixture was then cooled to room temperature. The solvent was removed in vacuo. The precipitate was redissolved in CH_2_Cl_2_. The resulting suspension was filtered off. The filtrate was concentrated in vacuo. The products were recrystallized from petroleum ether.

Compound **7a:** Yield 37%. MALDI-MS (*m*/*z*): (calc.): 876, 48 (M+), (found): 878, 35 (M+H).

Compound **7b:** Yield 52%. MALDI-MS (*m*/*z*): (calc.): 818, 47 (M+), (found): 818,14 (M+).

#### 3.1.5. Synthesis of DPI-Histidine-NHC-C-AuCl and DPI-Histamine-NHC-C-AuCl Complexes (**8a** and **8b**)

DPI (0.09 mmol) was dissolved in CH_2_Cl_2_, then EDC (0.135 mmol) and NHS (0.234 mmol) were added to the solution with stirring at 0 °C. After activation for 1 h, complex **4a** or **4b** (0.45 mmol) was added to the mixture with stirring. The reaction was carried out for 36 h under an argon atmosphere. The solvent was removed in vacuo. The products were obtained by TLC with CH_2_Cl_2:_ MeOH 10:1.

Compound **8a**: Yield 25%, MALDI-MS (*m*/*z*): (calc.): 1106.40 (M+), (found): 1108.62 (M+).

Calc. for C_48_H_61_AuClN_9_O_7_(%): C 52.01; H 5.55; N 11.37; Au 17.77; O 10.10; Cl 3.30. Found (%):C 52.06; H 5.58; N 11.39; Au 17.71; O 10.09; Cl 3.27.

Compound **8b**: Yield 32%, MALDI-MS (*m*/*z*): (calc.):1049.17 (M+) (found): 1049.42 (M+).

Calc. for C_46_H_59_AuClN_9_O_5_ (%): C 52.60; H 5.66; N 12.00; Au 18.75; O 7.62; Cl 3.38. Found (%):C 52.62; H 5.68; N 12.01; Au 18.71; O 7.63; Cl 3.36.

### 3.2. Biology

#### 3.2.1. Photoinduced Toxicity and Cytotoxicity Studies In Vitro

The human cell cultures used in the experiments included prostate adenocarcinoma (PC-3) and colorectal carcinoma (HCt116). Tumor cells were incubated in plastic flasks with a cell growth surface of 25 cm^2^ (Costar, Washington, DC, USA) on RPMI-1640 (PC-3 cell culture) and Eagle media (HCt116) with L-glutamine supplemented with 10% fetal calf serum (FBS) (PanEco, Moscow, Russia). Cells were incubated at 37 °C in a humidified atmosphere containing 5% CO_2_ (Binder CO_2_ incubator, Tuttlingen, Germany). Cell lines from 3 to 7 passages were used. In the experiments on assessment of the photoinduced activity of compounds, cells were cultured into 96-well culture plates at a concentration of 10^5^ cells per ml (where they were incubated for 24 h after exposure to light) or 7 × 10^4^ cells per ml (where they were incubated for 48 and 72 h after irradiation). Next, solutions of compounds were added to a final concentration ranging from 0.03 μM to 29 μM. After 4 h of incubation with photosensitizers, cells were irradiated with a halogen lamp through a KS-19 broadband filter (λ ≥ 720 nm). The power density was 19.5 ± 1.0 mW/cm^2^, and the calculated light dose was 10 J/cm^2^. After irradiation was completed, the plates with the cells were placed in a CO_2_ incubator for 24, 48, or 72 h, respectively. To estimate the cytotoxic activity, cells were incubated with compounds in the dark for selected time periods. Cell survival was estimated using a colorimetric MTT test. Based on the results, IC_50_ values were calculated, i.e., the PS concentrations that provided 50% cell death. Quantitative parameters were calculated from the results of three independent tests.

#### 3.2.2. In Vivo

Specific pathogen-free (SPF) BALB/c nu/nu, male, 8–12-week-old mice were obtained from the Russian National Center for Genetic Resources of Laboratory Animals at the Institute of Cytology and Genetics, Siberian Branch, Russian Academy of Sciences. All the animals were admitted with a veterinary passport and a certificate of quality. All procedures for routine animal care were performed in accordance with standard operational procedures and the sanitary rules for the design, equipment, and maintenance of experimental biological clinics and the Laboratory Animals manual [35,36].

Human prostate adenocarcinoma PC-3 was used as the tumor model. To perform implantation, a cell suspension was injected into animals in an amount of 7 × 10^6^ cells/mouse in the culture medium. To perform the experiments, PC-3 cells were inoculated in nu/nu mice, males, in an amount of 0.1 mL subcutaneously into the groin area on the inner side of the thigh.

The study of the biodistribution and estimation of fluorescence contrast of the compounds (2.0 mg/kg dose based on dipropoxy-BPI (**5**), single intravenous administration) were performed ex vivo in mice with PC-3 tumor (110 ± 10 mm^3^—day 7 of growth). Local fluorescence spectroscopy (LFS) was used to quantitatively determine the PS content in the tumor and in the surrounding skin. The level of normalized fluorescence that reflects the accumulation of a photoactive PS form in mice tissues was estimated with a “LESA-01” laser unit for fluorescence diagnostics and PDT control (BIOSPEK, Biospek, Russia).

At different time intervals after the photosensitizer injection (5, 15 min, 2, 4, 6, 14, and 24 h), animals were placed in a CO_2_ euthanasia box (ZOONLAB GmbH, Castrop-Rauxel, Germany), then tumor and skin tissue samples were extracted to evaluate their fluorescence. Exogenous fluorescence was measured ex vivo immediately after the euthanasia of an animal by the contact method. Normalized fluorescence (NF) was estimated at the wavelength matching the fluorescence peak of the PS, i.e., 811 ± 2 nm. Fluorescence was excited in the red region of the spectrum (λ = 633 nm). Mathematical processing of fluorescence spectra was performed using LESA-01 software 01-BIOSPEC. The integral intensity of PS fluorescence in the tissue (S_1_) was normalized to the integral signal intensity of diffuse backscattering of the excitation laser radiation (S_2_). Normalized PS fluorescence in biological samples was determined as the ratio of areas under the S_1_/S_2_ curve [37,38]. The fluorescence contrast (FC) of the PS in the tumor tissue was calculated with respect to the surrounding skin.

The photoinduced antitumor activity of the compounds was studied in mice with PC-3 adenocarcinoma xenografts (110 ± 8 mm^3^—day 7 of growth). The dose of the studied compounds was chosen as 1.0 mg/kg (based on dipropoxy-BPI (**5**)) at the stage of preliminary studies and administered once intravenously. The time interval before irradiation was determined based on biodistribution and fluorescence contrast results and amounted to 15 min. Surface irradiation was performed using an ALHT-ELOMED laser device for PDT (Russia) with an emission maximum of 810 nm, 4 W, corresponding to the absorption region of the compounds studied. Irradiation modes: power density Ps = 100 mW/cm^2^, energy density E = 90 J/cm^2^. Control animals were injected with 0.9% NaCl or compounds being studied without irradiation. The tumor of an animal was irradiated with a single light beam with a diameter of 10 mm, which completely covered the tumor and the surrounding tissue at 1–2 mm. Five to seven minutes before irradiation, an animal was anesthetized by injecting 0.25% droperidol solution intraperitoneally at a dose of 0.1 mL per mouse. Clinical examination of the animals was performed 2 h after the treatment and every 2–3 days thereafter. The tumor size was recorded for 21 days, and the tumor volume was calculated using the formula: V = a × b × c × 0.52, where a, b, and c are three mutually perpendicular tumor diameters. The observation was continued for 30 days after the treatment, then the animals were euthanized, and the presence or absence of the tumor mass was assessed macroscopically. The absence of a tumor node in 30 days after the treatment was considered as remission. The criteria for efficacy assessment included: inhibition of tumor growth (ITG) and the number of remissions (RN) [39]. A PS was considered highly efficient if one of the criteria was met: ITG ≥ 70%, RN = 50–100%.

The group arithmetic mean (M) and standard error of the mean (m) for all the quantitative values were calculated and presented in the tables and figures. The differences between the experimental and control groups were considered statistically significant at *p* ≤ 0.05.

## 4. Conclusions

In this study, gold(I) complexes based on NHC derivatives of histidine and histamine and their conjugates with derivatives of natural bacteriochlorophyll *a* were obtained. Two ways of the synthesis were implemented, which involve assemblage of the complex on a PS molecule or biding the metal complex based on an NHC-ligand to a bacteriochlorin macrocycle. The former approach made it possible to synthesize PS conjugates with NHC-derivatives of histidine and histamine, which were studied in vitro and in vivo; however, it proved to be inefficient for synthesizing metal complexes since the PS was subject to oxidation with Au(I) contained in (CH_3_)_2_SAuCl. The conjugates of PS with NHC derivatives of histidine and histamine were obtained by binding complexes of gold(I) with NHC derivatives of histidine and histamine to bacteriopurpurinimide.

In vitro biological tests on tumor cells from two cultures with hyper-expression and weak expression of histamine receptors (PC-3 and HCt116, respectively) showed that the C-donor Au(I) complexes (compounds **8a** and **8b**) exhibited the highest activity, both under light exposure and without irradiation. In vivo studies in mice with PC-3 prostatic adenocarcinoma xenografts revealed the accumulation in tumor tissue and high antitumor activity of the Au(I) metal complex of NHC-derived histamine with bacteriopurprinimide (ITG = 100%, RN = 100%) exceeding that of dipropoxy-BPI and its NHC derivative with histamine.

## Figures and Tables

**Table 1:** The photoinduced activity of compounds in the system in vitro.

Compounds	PC-3	HCt116
	IC_50_, nM	
**6a**	116 ± 11	125 ±12
**6b**	95 ± 20	89 ± 13
**7a**	98 ± 14	114 ± 22
**7b**	92 ± 10	110 ± 11
**8a**	140 ± 11	121 ± 20
**8b**	101 ± 11	96 ± 14
**b**	143 ± 20	100 ± 15

**Table 2:** Cytotoxic activity of compounds in vitro.

Compounds	PC-3			HCt116		
	Incubation Time of Compounds with Cells, Hours					
	24	48	72	24	48	72
	IC_50_, nM					
**6a**	6018 ± 301	5665 ± 108	4835 ± 105	6721 ± 202	5740 ± 311	4671 ± 115
**6b**	5834 ± 225	4315 ± 212	4202 ± 114	9634 ± 312	7220 ± 401	5708 ± 325
**7a**	5151 ± 157	4690 ± 241	5019 ± 228	6981 ± 300	6417 ± 321	5794 ± 201
**7b**	7085 ± 304	6477 ± 301	5580 ± 301	7813 ± 228	6721 ± 207	5980 ± 100
**8a**	6230 ± 165	5140 ± 118	3343 ± 298	5870 ± 199	4873 ± 210	2809 ± 117
**8b**	5834 ± 145	4382 ± 214	2331 ± 118	6572 ± 203	5437 ± 111	3145 ± 110
**5**	9635 ± 209	7907 ± 230	7044 ± 314	5887 ± 222	5655 ± 112	4712 ± 208

## Data Availability

The data presented in this study are available from the authors on request.
